# High ambient temperature and mortality: a review of epidemiologic studies from 2001 to 2008

**DOI:** 10.1186/1476-069X-8-40

**Published:** 2009-09-16

**Authors:** Rupa Basu

**Affiliations:** 1 California Office of Environmental Hazard Assessment, Air Pollution Epidemiology Section, Oakland, California, USA

## Abstract

**Background:**

This review examines recent evidence on mortality from elevated ambient temperature for studies published from January 2001 to December 2008.

**Methods:**

PubMed was used to search for the following keywords: temperature, apparent temperature, heat, heat index, and mortality. The search was limited to the English language and epidemiologic studies. Studies that reported mortality counts or excess deaths following heat waves were excluded so that the focus remained on general ambient temperature and mortality in a variety of locations. Studies focusing on cold temperature effects were also excluded.

**Results:**

Thirty-six total studies were presented in three tables: 1) elevated ambient temperature and mortality; 2) air pollutants as confounders and/or effect modifiers of the elevated ambient temperature and mortality association; and 3) vulnerable subgroups of the elevated ambient temperature-mortality association. The evidence suggests that particulate matter with less than 10 um in aerodynamic diameter and ozone may confound the association, while ozone was an effect modifier in the warmer months in some locations. Nonetheless, the independent effect of temperature and mortality was withheld. Elevated temperature was associated with increased risk for those dying from cardiovascular, respiratory, cerebrovascular, and some specific cardiovascular diseases, such as ischemic heart disease, congestive heart failure, and myocardial infarction. Vulnerable subgroups also included: Black racial/ethnic group, women, those with lower socioeconomic status, and several age groups, particularly the elderly over 65 years of age as well as infants and young children.

**Conclusion:**

Many of these outcomes and vulnerable subgroups have only been identified in recent studies and varied by location and study population. Thus, region-specific policies, especially in urban areas, are vital to the mitigation of heat-related deaths.

## Background

Although many studies of temperature have been conducted in other disciplines such as climatology, they have received greater attention in epidemiology in the past few years. In 2002, a comprehensive epidemiologic review by Basu and Samet [1] summarized the findings from studies examining mortality from elevated ambient temperature and heat waves. Most of the evidence at that time was based on studies following heat waves. Several studies have been conducted more recently using modern statistical approaches, consisting primarily of the time-series and case-crossover approaches. While other reviews have been conducted more recently on heat and mortality, the focus has been on methodological issues and approaches [2] and on climatology [3], leaving a number of important epidemiologic studies excluded. Many studies of ambient temperature and mortality did not account for air pollutants, and in the previous review [1], it was not clear from the few studies conducted whether air pollutants acted as confounders, effect modifiers, or both. It is critical to separate the independent effects of both ambient temperature and air pollutants, since they may often influence each other on a daily basis. Thus, the actual association between ambient temperature and mortality can be observed, only after accounting for pollutants in the models with ambient temperature. Furthermore, demographic characteristics, such as poverty and age, can modify the severity of heat effects through various physiological and behavioral pathways. Thus, certain subgroups may be particularly vulnerable to heat effects, and identifying them for specific locations would be beneficial for targeting public health interventions.

Here, the epidemiologic evidence from the past decade of high ambient temperature and mortality is summarized, with a closer examination of studies of the potential effect of air pollution on the temperature-mortality association, as well as vulnerable subgroups. A general discussion of mortality displacement on the association between temperature and mortality is also included. Mortality displacement (also known as harvesting) refers to the phenomena suggesting that observed deaths from some environmental exposure, such as ambient temperature, occur in the most frail individuals whose deaths have only been brought forward by a few days.

## Methodological issues

### Inclusion/exclusion criteria

All studies included in this review were published in peer-reviewed journals between January 2001 and December 2008. PubMed was used to search for the following keywords: temperature, apparent temperature, heat, heat index, and mortality. In addition, Table 2 had the keyword "air pollutants," "ozone," or "particulate matter" added, and Table 3 had "vulnerable", "susceptible subgroups/groups" added. The search was limited to the English language and epidemiologic studies. The review focused primarily on quantitative studies of ambient temperature, consisting of studies using the time-series and/or case-crossover methods. The outcomes from these studies generally reported a regression coefficient, relative risk (time series), odds ratio (case-crossover), or percent change in mortality, along with corresponding standard errors or confidence intervals. Studies that reported mortality counts or excess deaths following heat waves were excluded so that the focus remained on general ambient temperature exposure, rather than on short time periods. Review articles or studies focusing on cold temperature effects were also excluded. Thirty-six studies published in peer-reviewed journals were selected for this epidemiologic review, with 52 total studies in the references that included a general discussion of temperature and mortality.

### Exposure assessment

Temperature data are often measured near airport monitoring stations, and analyzed at the city or county level. Thus, misclassification of exposure may occur, especially for larger geographic areas. Also, measuring ambient temperature outside of urban areas may artificially reduce the temperature measurement, since urban areas often have higher temperatures than suburban or surrounding areas because of heat absorbed by buildings and roadways (known as the urban heat-island effect). However, since the bias should be non-differential (i.e., not different by county or other unit of analysis), the bias in the estimate would be toward the null, where the results would be underestimated. Misclassification of exposure may be reduced in future studies by using smaller buffer zones, such as five or ten kilometers around each monitor, if sufficient data are available.

Exposure to ambient temperature is often defined as some combined metric of temperature and relative humidity or dew point temperature, such as heat index, humidex, or apparent temperature, depending on the study location and author's preference. In addition, other variables, such as day of the week, time trend, and barometric pressure, are often added to the model. In some studies, air pollutants have been assessed as confounders or effect modifiers in an attempt to tease apart the independent effect of temperature. A monitor or an average of monitors is often used to characterize exposure for a county, or a given distance from the home address using geospatial coding. Many investigators relied on mean daily average to classify exposure, although others used maximum or minimum temperature to capture daytime and nighttime exposures, respectively, since those have also been shown to play a role in heat-related mortality. Because the effect of temperature has been found to be immediate (i.e., same lagged day), exposure can be characterized by the place of death.

### Case selection

Heat-related mortality is often underestimated, and since a systematic definition still does not exist, it may only be indicated when heat waves occur, resulting in a signal detection bias. Thus, investigators often use all-cause mortality excluding mortality due to accidents, or other related outcome, such as mortality from cardiovascular or respiratory diseases in epidemiologic studies of heat or elevated ambient temperature. The underlying cause of death is usually used for epidemiologic studies and should be sufficient for characterizing the temperature-mortality association, although associated causes of death can also be used.

The studies to date are often limited by information provided by the death certificate data. For example, information on income level, poverty, or air conditioning use is not offered on the individual level, so it is difficult to examine socioeconomic status. Thus, gathering data on individual characteristics, as has been done in a previous study [4], would be informative. In addition, medication use, time-activity patterns, and biologic mechanisms could be further understood.

### Study design

Most of the studies conducted in the past decade rely on the time-series or case-crossover study designs, with the exception of the studies of the 2003 European heat wave, which are not included in this review. Regardless of the method chosen, the time-series and case-crossover study designs should yield similar results, as has been shown in some temperature-mortality studies [5-7].

The time-series is a widely accepted approach in both air pollution and temperature studies. The time-series study generally encompasses large populations in multiple geographic areas over a given time period. Mortality counts or rates are compared to exposure measurements collected at regular time intervals (e.g., daily, weekly). Seasonality and other confounding factors that fluctuate over time are accounted for by adding the covariates to the model (i.e., day of the week, air pollutants) and using smoothing functions to the model using specified degrees of freedom.

The case-crossover design has been employed more recently, gaining widespread popularity for studying air pollution and temperature in the past decade. This study design is similar to a matched case-control study; however, each case in the case-crossover study serves as his/her own control. Thus, biases due to measured and unmeasured confounders, such as genetics, health behaviors, and physiologic differences, are controlled for by study design. The case-crossover study design has been refined since its introduction in 1991, from the unidirectional, to the bidirectional, and most recently, to the time-stratified approach. The time-stratified approach limits the bias from selecting control periods only previously to the case period (unidirectional), or from not selecting control periods at random from the time at which the case occurred (bidirectional). Most commonly, control periods are selected within the same month and the same year that the case period occurred in the time-stratified approach to inherently minimize biases that may occur from time trends. Day of the week is also matched for by study design by choosing control periods every seven days, or may be added to the model as an indicator variable, especially if data are sparse (e.g., if using particulate matter in the model, data are often collected every third day, and thus, would warrant control periods to be selected every third day). In the studies listed in the following tables, all case-crossover studies used the time-stratified approach.

Multi-city analyses are preferred, since bias from a single city analysis may result and the findings from multiple areas may be more generalizable [6]. Thus, city or county-level estimates are usually combined into one overall estimate using meta-analytic techniques using a random effects model.

### Mortality displacement

In epidemiologic studies of temperature or air pollution and mortality or morbidity, mortality displacement/harvesting have been addressed using several methods [8-13]. Among the more intuitive approaches, one can examine very long cumulative averages (i.e., 20 to 40 days of exposure) to determine whether a positive association found over the first few days is offset by a negative association over subsequent days. This would suggest that a pool of frail individuals was the only or major subgroup that was impacted by the exposure. However, if harvesting is not found, then the exposure under study is a real public health issue.

In studies of temperature and mortality, very few studies have addressed the harvesting issue. The evidence is mixed and may depend on: (1) whether one is examining heat waves versus a more general rise in temperature; (2) the study design and lag structure used for temperature effects; (3) the potential interactions with air pollution; (4) the baseline health status of the population; (5) the population at risk; and (6) other local factors that might determine vulnerability.

### Summary of Studies

Fourteen studies were epidemiologic studies of ambient temperature and mortality, while 14 other studies considered air pollutants as potential confounders/effect modifiers, and six considered vulnerable subgroups. Most of these studies used either the time-series method (n = 29), while fewer used the case-crossover approach (n = 10). Eleven studies were conducted in the US. Ten studies were published using European data, three in Latin America, three in Australia, two in Canada, and elsewhere. The studies are all summarized in the following Tables 1 to 3 by year of publication, with the most recent studies first, followed by alphabetical order of the first author's last name. Since some studies included an examination of general ambient temperature and mortality, accounted for air pollutants, and/or identified vulnerable subgroups, the same study may be listed in multiple tables with the relevant results.

### General Ambient Temperature and Mortality

In Table 1, the recent studies of high ambient temperature and mortality are summarized. To focus on the effects of warmer temperatures, most investigators limited their data above a threshold value, or have compared effect estimates from temperatures above a threshold value to another lower value. The threshold value is often based on some percentile of the data (i.e., 90^th ^or 95^th ^percentile), after visual inspection of the exposure-response curves or by mathematical (i.e., through derivatives) or statistical (i.e., by maximum likelihood) methods. The data are often limited to the summer months or warm season to establish heat effects. Limiting the data to summer months or the warm season is also employed to exclude possible (negative or positive) effects from cold temperatures on mortality in the attempt to estimate the actual risk from heat effects. Because of these several classifications of temperature exposure, it is difficult to directly compare the values resulting from these studies. However, few comparisons can be made. For example, in Europe and Korea, where different levels of temperature and humidity were experienced, the mortality estimates above a threshold of (23.3-29.7°C) resulted in different effect estimates [12]. With similar threshold values in the Mediterranean (29.4°C) and Korea (27-29.7°C), a 1°C increase of apparent temperature corresponded to a 3.12% increase in daily mortality in Mediterranean cities, and a much higher effect in Korea (6.73%-16.3% in six cities) for a similar time period. Two recent studies conducted by Basu et al. [6,14] and Zanobetti and Schwartz [7] using identical methods suggested that the effect estimates throughout California and other parts of the US are similar, even with different ranges of apparent temperature. They both found approximately a 2% increase in mortality associated with a 10°F increase in apparent temperature.

**Table 1:** Recent Studies of High Ambient Temperature and All-Cause Daily Mortality*

Reference	Study population	Method	Exposure	Result: effect estimate (95% CI)
Baccini 2008 [12]	15 European cities, April-September 1990-2000 (5-11 years depending on data availability for city)	Time-series; % change	Maximum apparent temperature (threshold 29.4°C Mediterranean cities and 23.3°C north-continental cities)	1°C increase above threshold 3.12 (0.60-5.72) in Mediterranean and 1.84 (0.06-3.64) in north-continental region Lag: 3 days prior

Basu 2008 [6]	9 California counties, May to September 1999-2003	Time-series and case-crossover; % change	Daily apparent temperature (minimum, mean, maximum); daily mean O_3_, PM_2.5_, PM_10_, NO_2_, CO, SO_2_	Per 10°F increase mean temperature, 2.3 (1.0-3.6), similar results for minimum and maximum temperatures Lag: 0

Bell 2008 [15]	Sao Paulo, Brazil, Santiago, Chile and Mexico City, Mexico, 1998-2002	Case-crossover; % change	Same day apparent temperature compared with days at 75^th ^percentile, O_3_, PM_10_	2.69 (-2.06, 7.88) for Santiago, 6.51% (3.57, 9.52) for Sao Paulo and 3.22% (0.93, 5.57) for Mexico City Lag: 0

McMichael 2008 [45]	Delhi, Monterrey, Mexico City, Chiang Mai, Bangkok, Salvador, Sao Paulo, Santiago, Cape Town, Ljubljana, Bucharest, Sofia, 2 to 5-year series (1991-1999)	Time-series; % change	Daily maximum threshold (16°C-31°C) temperature, relative humidity, precipitation data, PM_10_, BS, or TSP	1°C increase above threshold increased death rates with increasing heat in all cities: (ranging from 0.77-18.8) except Chiang Mai 2.39 (-0.49-5.35) and Cape Town 0.47 (-0.31-1.24) Lag: 2-day average

Vaneckova 2008a [46]	Sydney, Australia, October to March 1993-2001	Time-series; ratio of highest 10% mortality days within air mass and % frequency of air mass occurrence	Temporal Synoptic Index (TSI)	1.64 and 2.64 (both significant)for warmest TSIs, no CI provided

Zanobetti and Schwartz 2008 [7]	9 U.S. counties, May to September 1999-2002	Time-series and case-crossover; % change	Daily apparent temperature (minimum, mean, maximum); daily mean O_3_, PM_2.5_, PM_10_	Per 10°F increase mean temperature, 1.8 (1.09-2.5) case-crossover and 2.7 (2.0-3.5) time-series; similar results for minimum and maximum temperatures Lag: 0

Barnett 2007 [47]	107 U.S cities using data from the National Morbidity and Mortality Study, 1987-2000	Case-crossover; % change	Daily temperature	Per 10°F, summer 1987 average increase in cardiovascular deaths was 4.7 (3.0-6.5). By summer 2000, the risk with higher temperature had disappeared (-0.4, -3.2-2.5) Lag: 04

Medina-Ramon 2007 [21]	50 US cities in cold (November to March) and warm (May to September) seasons	Case-crossover; % change	Binary variable as extreme heat (range 22-32°C) and continuous; O_3_	5.74 (3.38-8.15) for extreme heat Lag: 2-day average

Kolb 2007 [32]	Montreal, Canada 1984-1993	Case-crossover; odds ratio	Mean daily and maximum temperature, barometric pressure, relative humidity, adjusted for O_3 _and both NO_2 _and O_3_	1.20 (1.14-1.38) for 25-30°C maximum temperature; strong nonlinear association with a threshold at 25°C Lag: average 02; no association after 3 days

Carson 2006 [48]	London, England, 4 time periods, winter: December-March; non-winter: April-November	Time-series; ratio of winter to non-winter deaths	Daily mean temperature	1.24 (1.16-1.34) from 1900-10,; 1.54 (1.42, 1.68) from 1927-37, 1.48 (1.35,-1.64) from 1954-64, 1.22 (1.13-1.31) from 1986-96; heat deaths diminished overall in the century

Kim 2006 [40]	6 cities in South Korea, summer 1994-2006	Time-series; % change	Daily mean temperature thresholds (27-29.7°C)	1°C above threshold 16.3 (14.2, 18.4), 9.10 (5.12, 13.2), 7.01 (4.42, 9.66), 6.73 (2.47, 11.2) for Seoul, Daegu, Incheon and Gwangiu, respectively

Michelozzi 2006 [49]	4 Italian cities, June to September 2003 & 2004 and reference period (Roma, Torino, Milano: 1995-2002 and Bologna: 1996-2002)	Time-series; % change	Daily maximum apparent temperature thresholds (28-32°C)	1°C above threshold 3.2 (1.9-4.6), 5.0 (3.8-6.1), 5.4 (4.3-6.5), 3.8 (2.5-5.0) for Bologna, Milano, Roma, and Torino, respectively

Stafoggia 2006 [16]	Bologna, Milan, Rome, Turin, 1997-2003	Case-crossover; odds ratio	30°C mean apparent temperature relative to 20°C; odds ratio	1.34 (1.27, 1.42) Lag: 01

Basu 2005 [5]	20 US metropolitan areas, seasonal analysis 1992	Time series (relative risk) and case-crossover (odds ratio)	Mean daily temperature per 10F adjusted for dew point temperature; daily O_3_	Per 10°F, 1.15 (1.07-1.24), 1.10 (0.96-1.27), 1.08 (0.92-1.26), 1.08 (1.02-1.15), and 1.01 (0.92-1.11) in the Southwest, Southeast, Northwest, Northeast, and Midwest, respectively, in the summer from the time-stratified case-crossover Lag: 0,1

El-Zein 2004 [34]	Greater Beirut, Lebanon, 1997-1999	Time-series; % change	Mean daily temperature, mean daily humidity, minimum mortality temperature (TMM) = 27.5°C	1°C above TMM 12.3 (5.7, 19.4%) increase in annual mortality Lag: 0

Goodman 2004 [26]	Dublin, Ireland, April 1980 to December 1996	Time-series; % change	Daily minimum temperature, daily mean relative humidity	1°C increase 0.4 (0.3-0.6) increase Lag: 0

Pattenden 2003 [50]	Sofia, Bulgaria (1996-1999) and London, England (1993-1996)	Time-series; % change	Daily mean temperature, relative humidity and PM (black smoke for London and total suspended particulates for Sofia)	1°C increase above 90th % 1.9 (1.4 to 2.4) in London, and 3.5 (2.2 to 4.8) in Sofia Lag: 2 day average

Curriero 2002 [39]	11 Eastern US cities, 1973-1994	Time-series; % change	Daily mean temperature, dew point temperature; minimum mortality temperature (MMT) range: 65.2-90.3	Per 10°F above MMT range 1.4-6.7 Lag: 0

Braga 2001 [11]	12 US cities. 1986-1993	Time-series; % increase	Mean daily temperature, relative humidity	4% increase (no CI given); Lag: 0 or 1 Harvesting effect for hot temperatures

* Exceptions: El-Zein 2004 and Carson 2006 reported annual and weekly deaths, respectively.

### Air Pollutants as Confounders/Effect Modifiers

Table 2 includes recent studies that have evaluated air pollutants as a potential confounder and/or effect modifier of the high ambient temperature and mortality association. The pollutants that have been examined include ozone (O_3_), particulate matter less than 10 μg/m3 in aerodynamic diameter (PM_10_), fine particulate matter (PM_2.5_), carbon monoxide (CO), sulfur dioxide (SO_2_), and nitrogen dioxide (NO_2_). Most investigators who considered pollutants evaluated PM and O_3_, since these pollutants have been found to be associated with mortality and are often correlated with high temperature.

**Table 2:** Recent Studies of High Ambient Temperature and Mortality Examining Air Pollutants as Potential Confounders and/or Effect Modifiers

Reference	Study location	Method	Exposure	Causes of death	Result
Basu 2008 [6]	9 California counties, May to September 1999-2003	Time-series and case-crossover	Same day mean apparent temperature; daily mean O_3_, also PM_2.5_, PM_10_, NO_2_, CO, SO_2_, lag 0 for PM, lag01 for gases	All-cause mortality	Confounders: none found Effect modifiers: none found

Bell 2008 [15]	Sao Paulo, Brazil, Santiago, Chile and Mexico City, Mexico, 1998-2002	Case-crossover	Same day apparent temperature compared with days at 75^th ^percentile, same day lag O_3_, same-day lag PM_10 _except Santiago lag 1 PM_10_	All-cause daily mortality	Confounders: O_3_, PM_10_ Effect modifiers: not studied

McMichael 2008 [45]	Delhi, Monterrey, Mexico City, Chiang Mai, Bangkok, Salvador, Sao Paulo, Santiago, Cape Town, Ljubljana, Bucharest, Sofia, 2 to 5-year series (1991-1999)	Time-series	Daily maximum threshold (16°C-31°C) temperature, relative humidity, precipitation data, PM_10_, BS, or TSP	All-cause mortality	Confounders: none found Effect modifiers: not studied

Ren 2008 [22]	US 95 NMMAPS counties, June to September 1987-2000	Time-series	Daily maximum temperature (same-day, lag 1), maximum hourly O_3_	CVD mortality	Confounders: not studied Effect modifier: O_3_

Vaneckova 2008a [46]	Sydney, Australia, October to March 1993-2001	Time-series	Temporal Synoptic Index (TSI) on the highest 10% mortality days, O_3_, PM_10_	All-cause, circulatory, cerebrovascular	Confounders: O_3 _on warm, humid days and PM_10 _on hot, dry days Effect modifiers: not studied

Vaneckova 2008b [19]	Sydney, Australia, October to March 1993-2004	Time-series	Daily maximum temperature, maximum O_3_	Underlying and associated causes of death	Confounders: O_3_, PM_10_ Effect modifiers: not studied

Zanobetti and Schwartz 2008 [7]	9 U.S. counties, May to September 1999-2002	Time series and case-crossover	Daily apparent temperature (minimum, mean, maximum); daily mean O_3_, PM_2.5_	All-cause mortality	Confounders: none found Effect modifiers: none found

Kolb 2007 [32]	Montreal, Canada 1984-1993	Case-crossover	Mean daily and maximum temperature, barometric pressure, relative humidity, adjusted for O_3 _and both NO_2 _and O_3_	Daily all-cause mortality	Confounders: none found Effect modifiers: not studied

Medina-Ramon 2007 [21]	50 US cities in cold (November to March) and warm (May to September) seasons	Case-crossover	Binary variable as extreme temperature and continuous; O_3_	All-cause and CVD mortality	Confounder: O_3_ Effect modifiers: not studied

Filleul 2006 [23]	9 French cities, all year and heat wave August 2003	Time-series	Minimum and maximum temperature, 8-hour maximum O_3_	Daily all-cause mortality	Confounders: not studied Effect modifier: O_3 _for some cities

Ren 2006 [51]	Brisbane, Australia (all year January 1996 to December 2001)	Time-series	Minimum temperature, daily PM_10 _as modifier	Cardiorespiratory mortality	Confounders: not studied Effect modifier: PM_10_

Stafoggia 2006 [16]	Bologna, Milan, Rome, Turin, 1997-2003	Case-crossover	30°C mean apparent temperature (lag01) relative to 20°C, O_3_	All-cause mortality	Confounders: none found Effect modifiers: not studied

Basu 2005 [5]	20 US metropolitan areas, seasonal analysis 1992	Time series (and case-crossover	Mean daily temperature per 10°F adjusted for dew point temperature; daily O_3_	Individual and daily cardiorespiratory mortality	Confounders: PM_10 _in summer Effect modifiers: not studied

O'Neill 2005 [18]	Mexico City (1996-98) and Monterrey (1996-99)	Time series; % change	Heat (35-36°C for Monterrey), mean temperature (25°C Monterrey, 15°C Mexico City), daily O_3_	Daily all-cause mortality	Confounders: O_3 _and PM_10 _on hot days Effect modifiers: not studied

Rainham and Smoyer-Tomic 2003 [17]	Toronto, May 1 to September 30, 1980-1996	Time-series; relative risk (RR)	Humidex, O_3_, also CO, NO_2_, SO_2_	Daily all-cause mortality	Confounders: none found Effect modifiers: not studied

Pattenden 2003 [49]	Sofia, Bulgaria (1996-1999) and London, England (1993-1996)	Time-series; % change	Daily weather (2-day mean) and PM (black smoke for London and total suspended particulates for Sofia)	Daily all-cause mortality	Confounders: none found Effect modifiers: not studied

Although the effect estimates changed with pollutants in the model, no significant confounding [15] or effect modification by pollution on the association between temperature and mortality was reported in some recent studies conducted in the US [6,7]. The studies conducted by Bell et al. and Zanobetti and Schwartz considered PM_10 _(as well as PM_2.5 _in the Zanobetti and Schwartz study) and O_3_, while the study by Basu et al. considered O_3_, PM_2.5_, PM_10_, NO_2_, CO, and SO_2_. Stafoggia [16] and Rainham and Smoyer-Tomic [17] also reported no confounding by O_3 _in Italy and Canada, respectively, and Pattenden did not find confounding by markers of PM in both Sofia (total suspended in particulates) and London (black smoke). However, PM_10 _was found to be confounder in Monterrey, Mexico [18], Sydney, Australia [19], and in regions throughout the United States, especially in the summer [5]. Ren and Tong [20] also observed PM_10 _to modify the association in their study conducted in Brisbane, Australia. O_3 _was found to be a confounder especially on hot days [18,21], and other investigators also showed O_3 _to be a positive effect modifier of temperature and mortality, at least in some study locations [22,23].

The results for confounding and/or effect modification by air pollutants on the temperature-mortality association remain mixed; as stated, some investigators reported air pollutants as confounders or effect modifiers while others found no significant confounding or effect modification in their studies.

### Cause-specific Outcomes and Vulnerable Subgroups

Much of the focus of epidemiologic studies has been identifying cause-specific outcomes and vulnerable subgroups of mortality from high ambient temperature (Table 3).

**Table 3:** Recent Studies Identifying Vulnerable Subgroups of Mortality from High Ambient Temperature

Reference	Study location	Study design	Exposure	Causes of death	Result
Baccini 2008 [12]	15 European cities, April-September 1990-2000 (5-11 years depending on data availability for city)	Time-series	Maximum apparent temperature (threshold 29.4°C Mediterranean cities and 23.3°C north-continental cities)	Daily all-cause mortality	Respiratory diseases among 75+ years

Basu and Ostro 2008 [14]	9 California counties, May to September 1999-2003	Case-crossover	Mean daily apparent temperature	Cause-specific mortality; all-cause mortality by age, race/ethnicity, gender, education level	Cardiovascular, higher specifically for ischemic heart disease, myocardial infarction, and congestive heart failure, ≤ 1 year, ≤ 5 years, elderly, Black race, out of hospital death; no elevated risks for cerebrovascular, diabetes, respiratory; no difference by gender or high school graduation

Bell 2008 [15]	Sao Paulo, Brazil, Santiago, Chile and Mexico City, Mexico, 1998-2002	Case-crossover	Same day apparent temperature	Daily all-cause mortality	65+ years, women in Mexico City, but men in Santiago and Sao Paulo, less educated in Sao Paulo

Ishigami 2008 [24]	Budapest, London and Milan, 2003	Time-series	Mean daily temperature (lag0 and lag1), PM_10 _(TSP in Budapest), ozone	Daily all-cause mortality	Increased age, females 65+ years greater risk in London and Milan and non-elderly adults in Milan; mortality from external causes, respiratory and cardiovascular diseases

Stafoggia 2008 [30]	4 Italian cities, 1997-2004	Case-crossover	Apparent temperature 30°C compared to 20°C	Deaths in hospitals for those with 2+ days in hospital	Increased age, single general medicine compared to high and intensive care units, history of psychiatric disorders, cerebrovascular diseases, heart failure, stroke, chronic pulmonary diseases

Vaneckova 2008a [46]	Sydney, Australia, October to March 1993-2001	Time-series	Temporal Synoptic Index (TSI); ratio of highest 10% mortality days within air mass and % frequency of air mass occurrence	Daily all-cause mortality	65+ years, women

Yip 2008 [52]	Maricopa County, Arizona, June to September 2000-2005	Time-series	Heat index	Heat-related deaths	Young and old outdoors, but greater risk for elderly indoors

Hajat 2007 [25]	England and Wales, 1993-2003	Time-series	Heat (> 95^th ^%) and cold (< 5^th ^%) thresholds	All-cause mortality	Elderly, those in nursing care homes respiratory and external causes, women; not modified by deprivation in London

Medina-Ramon 2007 [21]	50 US cities in cold (November to March) and warm (May to September) seasons	Case-crossover	Binary variable as extreme temperature and continuous; ozone	All-cause and CVD mortality	Cities with milder summers, less air conditioning and higher population density

Diaz 2006 [35]	Madrid, January 1986-December 1997	Time-series	T(hwave) = Tmax-36.5C if Tmax>36.5C; 5^th ^% to 95^th ^% temperature, NO_2_	AR = (RR-1)/RR for daily mortality	Circulatory causes, males 45-64 years

Stafoggia 2006 [16]	Bologna, Milan, Rome, Turin, 1997-2003	Case-crossover	30°C mean apparent temperature (lag01) relative to 20°C; odds ratio	All-cause mortality and previous hospitalization	Increased age and greater for women, widows and widowers, psychiatric disorders, depression, heart and circulatory disorders

Hajat 2005 [48]	Delhi, Sao Paulo, London, January 1991-December 1994	Time-series	Daily temperature (lag 0,1) greater than 20°C	Daily all-cause mortality	Respiratory deaths in Sao Paulo and London; children in Delhi

O'Neill, Zanobetti and Schwartz 2005 [37]	Chicago, Detroit, Minneapolis, Pittsburgh, 1988-1993 for Chicago and 1986-1993 for other cities	Time-series	Percent change daily mean temperature 29°C relative to 15°C (lag0), barometric pressure, day of the week, PM_10_	Mortality, prevalence of air conditioner (AC)	Black race, lack of air conditioner

Gouveia 2003 [33]	Sao Paulo, Brazil, 1991-1994	Time-series	Daily mean temperature (lag01), SO_2_, PM_10_, CO, O_3_, NO_2_, day of the week, season, humidity	Daily all-cause mortality, excluding violent deaths, cardiovascular and respiratory mortality	Greatest for 65+years and < 15 years, also increased for15-64 years; elderly cardiovascular, respiratory for adults and elderly; no modification by socioeconomic status

O'Neill 2003 [38]	7 US cities, 1986-1993	Time-series	Mean daily apparent temperature (% change 29°C and -5°C), PM_10_	Daily all-cause mortality, looking at effect modification by demographics & other variables	Black race, less educated, and outside hospital

Rainham and Smoyer-Tomic 2003 [42]	Toronto, May 1 to September 30, 1980-1996	Time-series	Humidex, CO, O_3_, NO_2_, SO_2_	Daily all-cause mortality	Females

Curriero 2002 [39]	11 Eastern US cities, 1973-1994	Time-series	Daily mean temperature, dew point temperature; minimum mortality temperature (MMT) range: 65.2-90.3	Daily all-cause mortality, excluding accidents	Higher latitude, more poverty, less air conditioning or heating

### Cause-specific outcomes

Some investigators have reported greater risks for deaths from cardiovascular (CVD) [5,14,24], respiratory [5,12,24-26], cerebrovascular [16], diabetes [27,28], or pre-existing psychiatric disorders [16,29,30]. Other studies also showed elevated risk from mortality subcategories of CVD diseases, such as myocardial infarction [8,14,31], ischemic heart disease [14], and congestive heart failure [14,30,32],

### Age

Age has been found to modify the association between ambient temperature and mortality. The elderly have been reported to be at greater risk from mortality following heat waves, as well as ambient temperature. In addition to the elderly who were at least 75 years [12], 70 years [31] or 65 years [5,14,15,19,24-26,33,34] of age, children under 15 years [18,33], children five years and younger [14], and infants one year of age and under [14,35] have been identified to be at increased risk for mortality from high ambient temperature. One investigator also reported 15 to 64 years of age to be at a significantly increased risk, although still lower than the elderly or young children [33].

### Gender

Modifications by gender has also been studied, and some investigators reported no difference by gender [14], while others found men in Santiago and Sao Paulo [15] specifically for circulatory causes [35] or women in various locations [15-17,19,24,36] to be at higher risk for mortality.

### Race/ethnic group

Other recent epidemiologic studies also reported Black racial ethnic group [14,37] and non-Whites [28] to be at greater risk than Whites in the US. Hispanic subgroups, however, have not been identified as being at greater risk in one study, partially explained by more social networking among this ethnic group [14].

### Socioeconomic factors

Other factors that provoked greater risk included indicators for lower socioeconomic status, including the less educated, persons living in lower income areas [16] and dying out of the hospital [14,27,38]. Also, increased poverty [39], and lack of air conditioner [11,37,39] were observed risk factors. However, lower socioeconomic status [33] and education level were not found to be a risk factor in all studies [14].

### Latitude Variations

Some studies reported variation by latitude, supporting the evidence for acclimatization. People who live in cities where the temperatures are generally elevated in the summer were found to have higher minimum mortality temperatures, or less risk given the same level of temperature, than people who live in cities with milder climates [7,21,39]. A similar finding was reported in California, where slightly higher estimates were found for coastal counties where milder temperatures are generally experienced [6]. Although coastal areas in California are usually more expensive, many of the homes lack air conditioning, since they have not been needed. Therefore, air conditioning prevalence is not an indicator of socioeconomic status in California, as it is in the remainder of the U.S.

## Discussion

In the past few years, several epidemiologic studies have been conducted in various locations to characterize temperature and mortality. In the US, similar effects were found in nine counties in California and in nine counties outside of California in two separate studies using the same methods [6,7]. In Europe and Korea, however, the effect estimates were larger [12,40], further supporting the need to conduct temperature-mortality studies for specific areas. The results from future studies can be more readily compared if estimates are reported per degree Celsius or Fahrenheit per unit change in temperature (assuming linearity), or if a regression coefficient is given, rather than selecting a threshold value for temperature. In addition, investigators should consider accounting for air pollutants and identifying vulnerable subgroups in their epidemiologic studies.

The recent epidemiologic evidence suggests that PM and O_3 _may be confounders, and some studies also found O_3 _to be an effect modifier in the warmer months. In other words, the association between temperature and mortality is partially a result of the effect of PM and O_3_. However, this confounding effect is relatively small, and there is clearly an independent effect of both temperature and air pollution on mortality. Others have reported that temperature has a greater effect on mortality with higher levels of O_3 _(i.e., synergism). Some of the conflicting evidence for confounding and effect modification by air pollutants may be due to high correlations between pollutants and temperature, making it difficult to tease apart the independent effects of either exposure. Also, different sources, chemistry, size distribution of particles, compositions and patterns of exposure [41] of gases and particles are observed throughout the US and elsewhere. Although O_3 _generally peaks in the summer throughout the US, for example, particulate matter peaks in the winter in California and in the summer on the East Coast. Thus, there would more likely be an impact of PM on elevated ambient temperature and health outcomes on the East Coast. Acclimatization may also play a critical role in the temperature-mortality association. People who live in areas where high ambient temperatures or heat waves are typically experienced may be less affected than people who reside in areas where high ambient temperatures are less commonly observed. Thus, even if there is effect modification between ambient temperature and a pollutant, such as O_3_, the influence on mortality may be minimal, but synergistic in areas where heat waves are uncommon.

Several vulnerable subgroups have been identified in the past decade of epidemiologic research, and were often dependent on study location and study population. Thus, region-specific policies, especially in urban areas, are vital to the mitigation of heat-related deaths. Specifically, those dying from cardiovascular, respiratory, and some specific cardiovascular diseases, such as ischemic heart disease, congestive heart failure, and myocardial infarction were at greater risk for heat-related mortality. Other vulnerable subgroups included: Black racial/ethnic group, women, those with lower socioeconomic status, and all age groups, particularly the elderly over 65 years of age as well as infants and young children.

Infants, young children, and the elderly should be specifically targeted in future studies to prevent heat-related mortality. With the elderly increasing in urban environments, an important research goal is the identification of clinical patterns of chronic diseases that increase the susceptibility to heat. Furthermore, vulnerable subgroups need to be further identified by cause-specific outcomes or demographics, such as racial/ethnic group. Furthermore, adverse birth outcomes have been found to be associated with air pollutants in previous studies, but have not been investigated, specifically for ambient temperature. Although previous studies of air pollution and birth outcomes have not accounted for temperature, some investigators have suggested seasonal associations, implying that temperature could also play a role with adverse birth outcomes and warrants further investigation.

Several biological mechanisms have been postulated for susceptible populations to heat-related mortality, particularly the elderly [42]. When body temperatures rise, blood flow generally shifts from the vital organs to underneath the skin's surface in an effort to cool down. The body's ability to regulate its temperature (also known as thermoregulation) may be impeded when too much blood is diverted, putting increased stress on the heart and lungs. Increased blood viscosity, elevated cholesterol levels associated with higher temperatures, and higher sweating threshold may also trigger heat-related mortality [43]. The body's ability to adapt to high ambient temperature can be influenced by acclimatization. People who live in areas where high ambient temperatures are not generally experienced are more likely to be affected by a heat wave. The synergistic impact of high ambient temperature along with high levels of air pollutants, such as O_3 _and PM, may also play a role in increasing the mortality effect. Furthermore, heat waves occurring earlier in the year may have a greater impact on mortality since the population has not had the chance to adapt to hotter temperatures.

This review is timely as climate change receives more global attention, and more epidemiologic studies have been recently conducted. It, however, has several limitations. While it includes the most recent epidemiologic studies using time-series and case-crossover methods, it does not include studies of heat waves or studies using other approaches in an effort to focus on general ambient temperature over longer time periods. Both methods rely on ecologic exposure variables for temperature, and the time-series analysis also uses aggregated counts of mortality. Thus, an advantage of the case-crossover study is that differences by individual-level characteristics such as age, race/ethnic group, gender can be analyzed. Although the methods used across studies were similar, it was still often difficult to compare estimates between studies because of the analysis type (e.g., different threshold values). There were also not a sufficient number of studies to conduct a meta-analysis of the results, or other more substantial quantification. Finally, there may be some publication bias in the studies that were chosen, but by using PubMed, the bias may be limited, as it includes most scientific journals.

Further studies need to be conducted in more urban locations so that policies can be implemented for specific areas rather than for an entire geographic area. These studies would be helpful to the National Weather Service, health care institutions, and governmental agencies to implement policies to prevent heat-related mortality and also create a better heat warning system based on current studies. They will also be helpful to establish policy guidelines for the U.S. Environmental Protection Agency (personal communication), and could be used for economic analyses. Although no formal evaluation of heat-health watch warning systems has been performed to date, some city-based heat-health watch warning systems have already been implemented appear to be successful in greatly reducing mortality following heat waves [44]. For example, the 2003 heat wave in Western Europe resulted in 35,000 deaths, but the World Health Organization's project, EuroHEAT, collected information about existing warning systems and defined guidelines for prevention so that subsequent heat waves do not produce such devastating results http://www.euro.who.int/document/e91347.pdf.

## Conclusion

In the past few years, several epidemiologic studies have been conducted in various locations to characterize temperature and mortality. These studies have consisted primarily of time-series and case-crossover studies, and were summarized this review. The recent epidemiologic evidence suggests that PM and O_3 _may be confounders, and some studies also found O_3 _to be an effect modifier in the warmer months. However, this confounding effect is relatively small, and there is clearly an independent effect of both temperature and air pollution on mortality. Several vulnerable subgroups have been identified, including those dying from cardiovascular, respiratory, and some specific cardiovascular diseases, such as ischemic heart disease, congestive heart failure, and myocardial infarction. Other vulnerable subgroups included: Black racial/ethnic group, women, those with lower socioeconomic status, and all age groups, particularly the elderly over 65 years of age as well as infants and young children.

Thus, infants, young children, and the elderly should be specifically targeted in future studies to prevent heat-related mortality. Many of these outcomes and vulnerable subgroups have only been identified in recent epidemiologic studies of ambient temperature and were dependent on the location and study population. Thus, region-specific policies, especially in urban areas, are vital to the mitigation of heat-related deaths.

## Abbreviations

CO: (carbon monoxide); NO_2_: (nitrogen dioxide); O_3_: (ozone); PM: (particulate matter); PM_2.5_: (particulate matter with less than 2.5 μg/m^3 ^in aerodynamic diameter); PM_10_: (particulate matter with less than 10 μg/m^3 ^in aerodynamic diameter); SO_2_: (sulfur dioxide); °C: (degree Celsius); °F: (degree Fahrenheit); CI: (confidence interval).

## Competing interests

The author declares that they have no competing interests.

## Authors' contributions

RB conducted the literature search for this review, specified the inclusion and exclusion criteria, constructed the tables, and drafted and revised the manuscript for consideration for publication.
